# High Initial Reversible Capacity and Long Life of Ternary SnO_2_-Co-carbon Nanocomposite Anodes for Lithium-Ion Batteries

**DOI:** 10.1007/s40820-019-0246-4

**Published:** 2019-03-01

**Authors:** Pan Deng, Jing Yang, Shengyang Li, Tian-E Fan, Hong-Hui Wu, Yun Mou, Hui Huang, Qiaobao Zhang, Dong-Liang Peng, Baihua Qu

**Affiliations:** 1 Pen-Tung Sah Institute of Micro-Nano Science and Technology, Department of Materials Science and Engineering, College of Materials, Xiamen University, Xiamen, 361005 People’s Republic of China; 2 College of Automation and Key Laboratory of Industrial Internet of Things and Networked Control, Ministry of Education, Chongqing University of Posts and Telecommunications, Chongqing, 400065 People’s Republic of China; 3 Department of Chemistry, University of Nebraska-Lincoln, Lincoln, NE 68588 USA; 4 School of Mechanical Science and Engineering, Huazhong University of Science and Technology, Wuhan, 430074 People’s Republic of China

**Keywords:** Ultrafine SnO_2_ nanostructures, ZIF-67 frameworks, Enhanced initial Coulombic efficiency, Reversible conversion reaction

## Abstract

The two major limitations in the application of SnO_2_ for lithium-ion battery (LIB) anodes are the large volume variations of SnO_2_ during repeated lithiation/delithiation processes and a large irreversible capacity loss during the first cycle, which can lead to a rapid capacity fade and unsatisfactory initial Coulombic efficiency (ICE). To overcome these limitations, we developed composites of ultrafine SnO_2_ nanoparticles and in situ formed Co(CoSn) nanocrystals embedded in an N-doped carbon matrix using a Co-based metal–organic framework (ZIF-67). The formed Co additives and structural advantages of the carbon-confined SnO_2_/Co nanocomposite effectively inhibited Sn coarsening in the lithiated SnO_2_ and mitigated its structural degradation while facilitating fast electronic transport and facile ionic diffusion. As a result, the electrodes demonstrated high ICE (82.2%), outstanding rate capability (~ 800 mAh g^−1^ at a high current density of 5 A g^−1^), and long-term cycling stability (~ 760 mAh g^−1^ after 400 cycles at a current density of 0.5 A g^−1^). This study will be helpful in developing high-performance Si (Sn)-based oxide, Sn/Sb-based sulfide, or selenide electrodes for LIBs. In addition, some metal organic frameworks similar to ZIF-67 can also be used as composite templates.

**Electronic supplementary material:**

The online version of this article (10.1007/s40820-019-0246-4) contains supplementary material, which is available to authorized users.

## Highlights

- SnO_2_-Co-carbon nanocomposites were in-situ prepared from Co-based metal–organic frameworks and showed a remarkably high initial Coulombic efficiency (82.2%) and a capacity of ~ 800 mAh g^−1^ at a high current density of 5 A g^−1^.
- Facile approach for designing highly reversible and stable electrodes for next-generation high-performance lithium-ion batteries.

**Electronic supplementary material:**

The online version of this article (10.1007/s40820-019-0246-4) contains supplementary material, which is available to authorized users.

## Graphical abstract

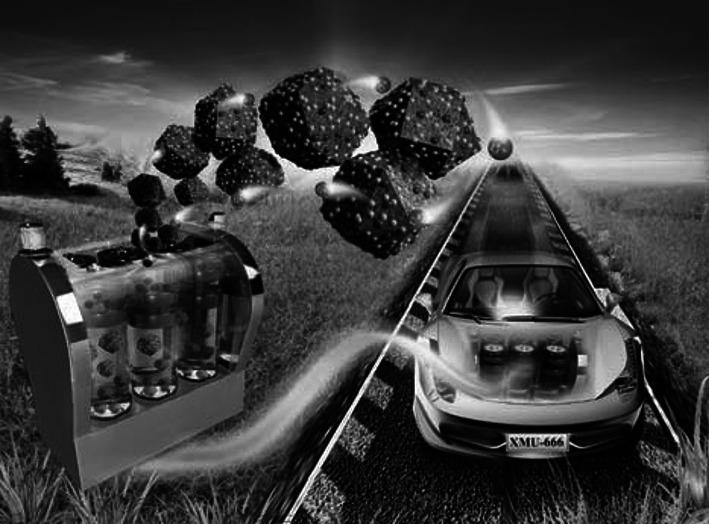

## Introduction

Over the past few years, the applications of lithium-ion batteries (LIBs) have extended from consumer electronics to power batteries. This impressive progress achieved in this field suggests that LIBs will continue to be a dominant power source for electric vehicles in the next decade [1]. In the pursuit of further improvement of LIBs, various efforts have been made to rationalize their design and to develop advanced electrode materials with high specific capacity, prolonged life span, and good rate capability [2, 3]. Graphite is the most widely used LIB anode. However, it exhibits a limited specific capacity of 372 mAh g^−1^. Therefore, in 2011, Sony Corporation produced novel LIBs (Nexelion). Specifically, their anodes consisted of Sn-Co–C composites. Various tin-based anodes have also been fabricated in order to develop high-performance LIBs [2–4]. Among these anodes, tin oxide (SnO_2_) anodes have been extensively studied because SnO_2_ can store Li^+^ via a two-step reaction and shows a high theoretical specific capacity of 1494 mAh g^−1^. In the Li^+^ storage of SnO_2_, the first step is the conversion reaction (SnO_2_ + 4Li → Sn + 2Li_2_O), which generates a capacity of 731 mAh g^−1^. In the subsequent lithiation/delithiation process, an alloying reaction (Sn + 4.4Li → Li_4.4_Sn) occurs, delivering a capacity of 763 mAh g^−1^ [6–8]. However, there are two major challenges in developing SnO_2_ anodes with a high specific capacity: (i) capacity loss induced by the huge volume variations (greater than 300%) generated during cycling, (ii) the irreversibility of the conversion reaction, which reduces the initial Coulombic efficiency (ICE) of the anode [5–7].

Various attempts have been made to overcome these limitations. For example, various carbon-based SnO_2_ composites have been developed to accommodate the volume expansions caused by cycling in order to achieve cycling stability [8–18]. However, this carbon composite approach cannot improve the ICE of SnO_2_ anodes. Hu et al. reported that SnO_2_ electrodes with Sn grains < 11 nm in diameter show a completely reversible conversion reaction (Sn + Li_2_O → SnO_2_) [19]. Transition metals (M = Cu, Fe, Mn, Co, etc.) or metal oxides are used to stabilize nanostructured SnO_2_ and can also improve the reversibility of the conversion reaction between Li_2_O and SnO_2_ [4, 20–25]. Inactive metals can buffer the expansion of Sn particles (to coarsen them) and migrate to the Sn/Li_2_O surface so that Sn can remain active with Li_2_O. Thus, the introduction of transition metals can improve the ICE of SnO_2_ electrodes. To sum up, there are mainly three mainstream solutions for developing high-performance SnO_2_-based anodes: (1) designing a unique carbon-based structure including the surface coating to suppress the full volume expansions [26, 27] while providing an expansion space [8, 28–31], (2) preparing ultrafine SnO_2_ nanoparticles to aggrandize the grain boundaries and alleviate the mechanical strain and improve the reversibility of the conversion reaction [6], and (3) introducing transition metals or forming intermetallic alloys to make the conversion reaction reversible and mitigate the expansion of Sn simultaneously [4, 20–23].

Recently, metal–organic frameworks (MOFs) with inorganic (Co and Zn) and organic molecules have been used as a novel 3D porous carbon source for developing adjustable templates to anchor guest transition metals [32–34]. In this study, we fabricated a novel ternary SnO_2_-Co-C composite by mixing ultrafine SnO_2_ nanoparticles with a Co-based MOF (ZIF-67) (denoted as N-u-SCC) to develop LIB anodes with high ICE and long-term cycle stability. ZIF-67 serves as a sacrificial template for the formation of Co additives and 3D porous carbon frameworks. This well-designed structure showed the advantages of the unique 3D carbon-based nanostructure with in situ formed Co additives and suppressed the volume expansions and Sn coarsening of the lithiated SnO_2_. This improved the cycling performance of the anodes and rendered the conversion reaction highly reversible. The 3D porous carbon framework served as an excellent carrier for SnO_2_ (to be anchored) and improved the conductivity of the entire composite while providing enough space for volume variations during the lithiation/delithiation process. The in situ formed Co additives not only prevented the covering of SnO_2_ by Li_2_O and alleviated the volume expansions, but also served as good electron conductors. As a result, the N-u-SCC-2 electrode showed a high ICE of 82.2% (average level). In addition, the electrodes showed extraordinary specific capacity (~ 975 mAh g^−1^ after 100 cycles at 0.2 A g^−1^), high capacity retention (78.6% after 100 cycles at 0.2 A g^−1^), excellent rate capability (a reversible capacity of ~ 800 mAh g^−1^ under the current density of 5 A g^−1^), and prolonged life span.

## Experimental Section

All the chemicals used in this work were analytically pure and were commercially available. Commercial SnO_2_ and Co(NO_3_)_2_·6H_2_O, 2-methylimidazole were purchased from Shanghai Macklin Biochemical Co. Ltd. Na_2_SnO_3_ and urea were purchased from Xilong Scientific.

### Synthesis of Ultrafine SnO_2_

Ultrafine SnO_2_ was prepared by modifying the method reported by Lou et al. [3]. In a typical reaction, 2.5 mmol of NaSnO_3_·4H_2_O and 16 mmol of urea were added into a solution of 145 mL of H_2_O and 15 mL of ethanol and the resulting mixture was stirred for 1 h. The reaction mixture was then transferred to a Teflon-lined stainless-steel autoclave, which was heated in an oven at 190 °C for 15 h. The reaction mixture was centrifuged to obtain precipitates, which were dried at 80 °C overnight and annealed at 550 °C for 4 h.

### Synthesis of ZIF-67 Frameworks

ZIF-67 frameworks were prepared by a simple liquid-phase method. Certain amounts of Co(NO_3_)_2_·6H_2_O (listed in Tables 1 and S1) (A) and 2-methylimidazole (B) were separately added into equal proportions of a methanol/ethanol solution under stirring, and the resulting solutions were labeled as solutions A and B, respectively. Solution B was quickly added to solution A, and the resulting mixture was vigorously stirred for another 3 min. The reaction mixture was then static aged for 22 h [33]. Precipitates were separated from the solution by centrifugation and were freeze-dried and then annealed at 550 °C for 2 h.

**Table 1:** Part of basic facts of the N-u-SCC composites

Defined name	Mole dosage of Co(NO_3_)_2_·6H_2_O (mmol)	Mole dosage of 2-Melm (mmol)	Carbon content (%)	ICE (%)
N-u-SCC-1	1.25	10	5.43	75.0
N-u-SCC-2	1.25	20	9.68	82.2

### Synthesis of N-c-SCC and N-u-SCC Composites

The N-doped commercial SnO_2_-Co-C (denoted as N-c-SCC) and N-u-SCC composites were prepared using a method similar to that used for the preparation of the ZIF-67 frameworks. The only difference was that 0.2 g of SnO_2_ (commercial or ultrafine) was added into solution A followed by sonication for 0.5 h. After the sonication, solution A was stirred for another 10 min for better dispersion. Then, solution B was quickly added to solution A and the resulting mixture was stirred vigorously for another 3 min. The reaction mixture was then static aged for 22 h. The precipitates separated from the solution by centrifugation were freeze-dried and then annealed at 550 °C for 2 h.

### Material Characterization

The morphology of the as-prepared samples was examined by a SUPRA 55 field-emission scanning electron microscope (FESEM). Transmission electron microscopy (TEM) examinations were carried out on a JEOL JEM 2100F at 200 kV. The elemental mapping and energy-dispersive X-ray spectroscopy (EDS) measurements of the samples were taken on an energy-dispersive X-ray spectrometer equipped with the JEOL 2100F microscope. The powder X-ray diffraction (XRD) patterns of the samples were recorded on a Rigaku Ultima IV with Cu Kα radiation (*λ* = 0.15418 nm). X-ray photoelectron spectroscopy (XPS) analysis was carried out using an Axis Ultra DLD spectrometer.

### Electrochemical Measurements

The electrochemical performance of the as-prepared composites was evaluated using R2032 coin-type half cells assembled in a glove box filled with argon. The oxygen and moisture contents of the glove box were < 0.5 ppm. The electrodes were prepared by confecting a slurry containing the active materials, carbon black, and carboxymethyl cellulose with a ratio of 7:2:1 in a solution of water and ethanol. This slurry solution was stirred for 8 h and was then casted onto a Cu foil and dried at 80 °C for 12 h under vacuum. The active material loading on each electrode was 0.9–1.1 mg. A solution of 1 M LiPF_6_ in ethylene carbonate and diethyl carbonate (at a volume ratio of 1:2) containing 10 wt% fluoroethylene carbonate (FEC) was used as the electrolyte. The electrochemical measurements of the electrodes were taken using a Neware battery tester over the potential range of 0.01–3.0 V. The specific capacity of the composites was calculated using their whole masses. Cyclic voltammetry (CV) measurements were taken on an electrochemical workstation (CHI 660C) over the voltage range of 0.01–3 V at a scan rate of 0.1 mV s^−1^.

## Results and Discussion

**Fig. 1:**
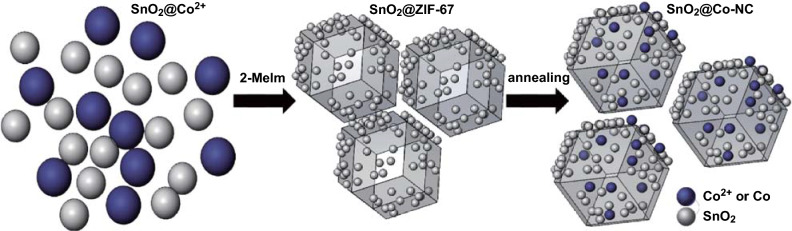
Schematic of the synthesis of N-u-SCC

Figure 1 shows the schematic of the synthesis of N-u-SCC. The as-prepared ultrafine SnO_2_ nanoparticles and Co(NO_3_)_2_·6H_2_O were uniformly dispersed in an alcoholic solution. To this solution, an alcoholic solution of 2-methylimidazole (2-Melm) was added to generate the SnO_2_@ZIF-67 composite. After annealing under an inert atmosphere, ZIF-67 carbonized and its structure collapsed. This eventually resulted in the formation of carbon frameworks with metallic Co. We also prepared composites using commercial SnO_2_. For this, commercial SnO_2_ was added to the alcoholic solution during the synthesis of ZIF-67. The reaction mixture was then heat-treated under an inert atmosphere to finally obtain the in situ formed N-c-SCC. Table S1 lists the synthetic formulae and ICE values of the three N-c-SCC electrodes. The morphology of commercial SnO_2_ and the three N-c-SCC composites is shown in Fig. S1.

**Fig. 2:**
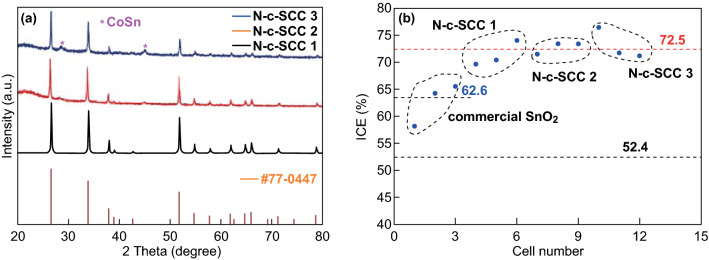
**a** XRD patterns of the three N-c-SCC (different ratio) samples. **b** ICEs of the different electrodes

The phase composition of the samples was analyzed using their XRD patterns. The XRD patterns shown in Fig. 2a reveal that SnO_2_ (JCPDS No. 77-0447) was the main phase of all the three samples. With an increase in the carbon content, the CoSn phase of the samples became predominant. No other impurity was detected. Figure 2b shows the ICE of the pure commercial SnO_2_ and N-c-SCC electrodes. The N-c-SCC electrodes showed better electrochemical performance than the pure commercial SnO_2_ electrode. The ICE of the N-c-SCC electrodes increased from 62.6 to 72.5%. This is because Co nanoparticles improved the reversibility of the conversion reaction on SnO_2_. Taking the variate into consideration, this remarkable improvement in the ICE of the N-c-SCC electrodes was due to the ZIF-67 frameworks, which consisted only of Co and carbon. This demonstrates the viability of our design, in which ZIF-67 was used as the sacrificial template. To further evaluate the electrochemical performance of the four electrodes, their charge–discharge curves were plotted, as shown in Fig. S2. The charging potential of all the SnO_2_ electrodes could be divided into three regions, each of which involved different reaction processes. Similarly, the capacity of the electrodes in each cycle could be divided into three parts: 0.01–1.0 V corresponding to the dealloying reaction (Li_*x*_Sn → Sn), 1.0–2.4 V corresponding to the conversion reaction (Sn → SnO_2_), and 2.4–3.0 V corresponding to some other reactions like the consumption of the electrolyte [25]. On the basis of the electrochemical measurements and morphological analysis of the electrodes, it can be stated that (1) the contact area between Co and SnO_2_ significantly improves the reversibility of the conversion reaction. At the N-u-SCC-1, the highest capacity was observed in all the cycles over the potential range of 1.0–2.4 V. (2) An increase in the carbon content improved the reversibility of the conversion reaction and the cycling stability of the electrodes. Figure S2 reveals that the N-c-SCC-3 electrode showed the best cycling stability.

**Fig. 3:**
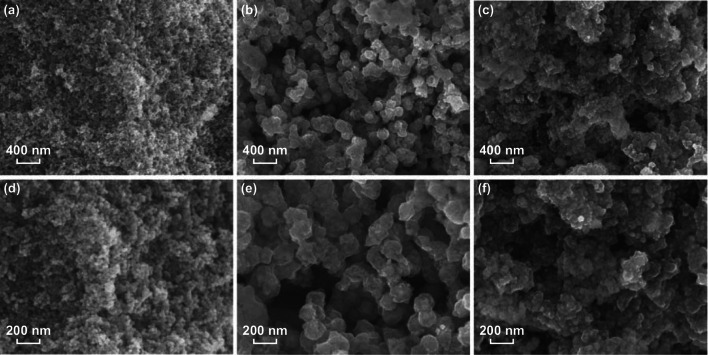
Morphology of **a**, **d** ultrafine SnO_2_; **b**, **e** N-u-SCC-1; **c**, **f** N-u-SCC-2

To further optimize the electrochemical performance of the SnO_2_ electrodes, commercial SnO_2_ was substituted by the as-prepared ultrafine SnO_2_ nanoparticles (~ 15 nm). The N-doped ultrafine SnO_2_-Co-C composites (denoted as N-u-SCC) were prepared using a method same as that used for preparing the N-c-SCC composites. Table 1 lists the synthetic formulae, carbon contents, and Sn and Co weight ratios of the two N-u-SCC composites. The morphology of the ultrafine SnO_2_ nanoparticles and N-u-SCC-1 and N-u-SCC-2 electrodes was observed. Figure 3a, d shows that the ultrafine SnO_2_ nanoparticles had a diameter of ~ 15 nm and were evenly distributed in the composites. The morphology and diameter of the frameworks of N-u-SCC-1 (Figs. 3b, e) were similar to those shown in Fig. S1c. This can be attributed to the same Co(NO_3_)_2_·6H_2_O/2-Melm ratio (1:8) in both the cases. Since the ultrafine SnO_2_ particles were much smaller than commercial SnO_2_ particles, they offered a larger contact area with metallic Co. At the Co(NO_3_)_2_·6H_2_O/2-Melm ratio of 1:16, the frameworks became less stable and could be hardly observed. However, carbon was predominant, as shown in Fig. 3c, f.

**Fig. 4:**
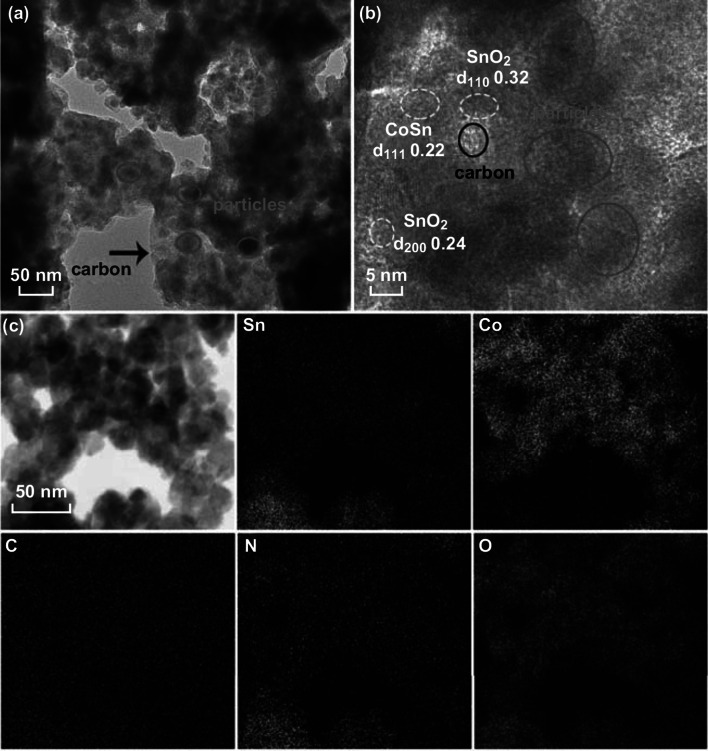
**a** TEM, **b** high-resolution TEM, and **c** elemental mapping images of the N-u-SCC-2 composites

The morphology of the N-u-SCC-2 composite was further analyzed by TEM. Figure 4a shows a typical TEM image of the as- prepared N-u-SCC-2 composite. It can be observed that the carbon frameworks consisted of SnO_2_ and Co (CoSn) nanoparticles (light zone), which alleviated the volume variations during the subsequent deintercalation reactions. The sufficient contact area between SnO_2_ and Co (CoSn) improved the reversibility of the conversion reaction, thus improving the ICE and cycling performance of the electrode. The lattice distances of 0.32 and 0.24 nm (Fig. 4b) correspond to the (110) and (200) planes of crystalline SnO_2_. The lattice distance of 0.22 nm corresponds to the (111) lattice plane of CoSn. The lattice distances of Sn could not be detected. This can be attributed to the low Sn content of the nanocomposite and the similarity in the lattice distances of Sn and SnO_2_. Figure 4c shows the elemental mapping of the N-u-SCC-2 composite under ultrahigh magnification. This figure confirms that Sn and Co were uniformly distributed in the carbon frameworks and showed sufficient contact. The Sn and Co weight ratios of N-u-SCC-2 were about 50.8% and 11.2%, respectively, while those of N-u-SCC-1 were 51.5% and 25.1%, respectively, as revealed by the EDS results (Table S2).

**Fig. 5:**
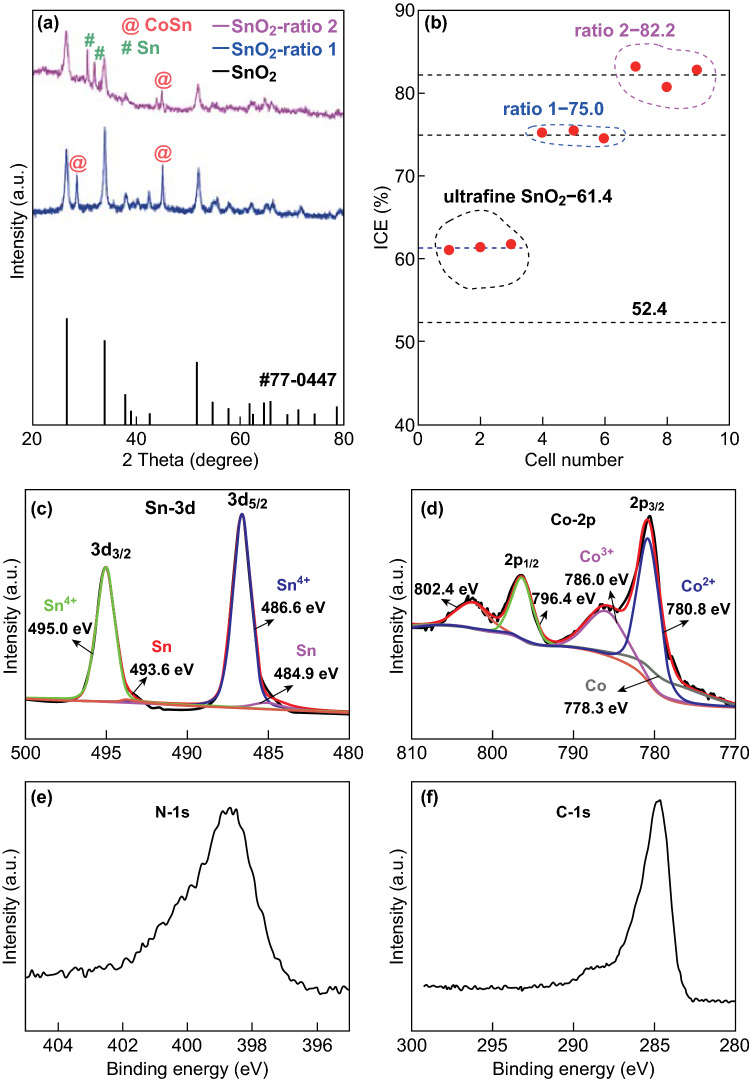
**a** XRD patterns of the three samples; **b** ICEs of the different electrodes. **c** Sn 3d, **d** Co 2*p*, **e** N 1*s,* and **f** C 1*s* XPS spectra of the N-u-SCC-2 composite

The phase composition of the ultrafine SnO_2_ particles and N-u-SCC-1 (SnO_2_-ratio 1 in Fig. 5a) and N-u-SCC-2 composites (SnO_2_-ratio 2 in Fig. 5a) was analyzed using their XRD patterns. The XRD peaks of ultrafine SnO_2_ could be indexed to JCPDS No. 77-0447. Like commercial SnO_2_, both N-u-SCC-1 and N-u-SCC-2 consisted of the CoSn phase, which was formed during the annealing process. This confirms the presence of Co in these composites. It should be noted that N-u-SCC-2 showed the Sn metal phase (Fig. 5a). This can be attributed to its stronger reduction capability owing to its higher carbon content (according to the EDS results; Tables 1 and S2). N-u-SCC-1 consisted mainly of the SnO_2_ phase with little CoSn. N-u-SCC-2 also consisted of SnO_2_ as the main phase with small fractions of the Sn and CoSn phases. In these composites, Co mainly existed as the CoSn phase. Figure 5b compares the ICE values of the N-u-SCC electrodes. The N-u-SCC-2 electrode showed the highest ICE value of about 82.2% (Fig. S4). N-u-SCC-2 showed a higher ICE than N-u-SCC-1 because of its larger Sn/Co contact area, as revealed by the FESEM images.

The valence states of Sn and Co in the N-u-SCC-2 composite were analyzed by XPS. As can be observed from Fig. 5c, Sn showed a main valence of Sn^4+^. This is consistent with the observation that SnO_2_ was the main phase of this composite. In addition, some Sn present on the surface showed a valence of zero. This can be attributed to the reduction of metallic Sn by carbon and Co during the annealing process (as observed from the XRD patterns). Oxygen vacancies were generated at this point. The Co 2*p* spectra (Fig. 5d) of the near-surface region of the composite showed three types of valence (Co, CoO, and Co_2_O_3_). The increasing valence could be assigned to the oxidation of metallic Co. The small amount of oxidation in the near surface of the composite could not be detected by XRD. Figure 5e, f shows the N 1*s* and C 1*s* spectra of the N-u-SCC-2 composite and confirms the presence of N and C in it. Moreover, the high-resolution N 1*s* XPS spectra showed that the composite consisted of pyridinic N (398.7 eV) and pyrrolic N (400.5 eV) (Fig. S5). The pyridinic N and pyrrolic N contents of this composite were 80.3% and 19.7%, respectively (Fig. S5). These results indicate the N-doped C composite SnO_2_ was the main active phase of the N-u-SCC-2 composite.

**Fig. 6:**
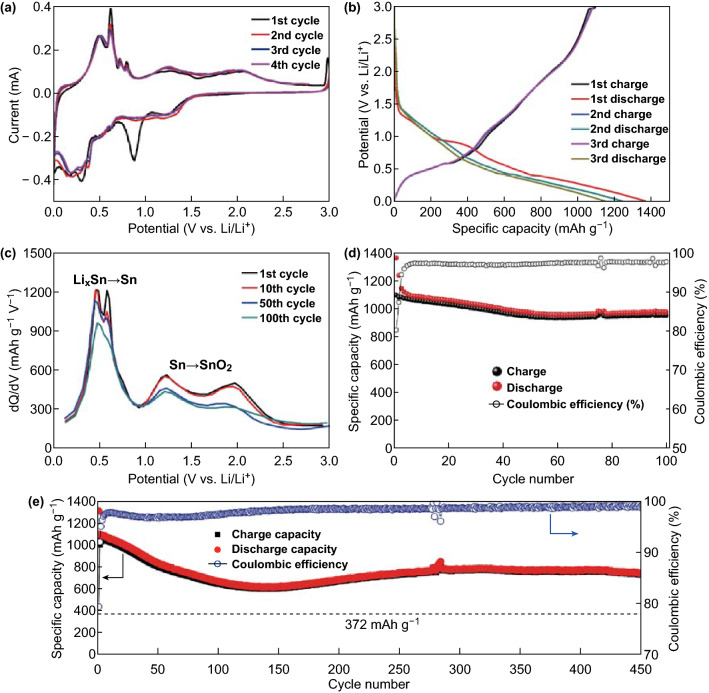
**a** CV curves of the N-u-SCC-2 electrode over the potential range of 0.01–3 V. **b** First three discharge/charge curves of the N-u-SCC-2 electrode at a current density of 0.2 A g^−1^. **c** Differential charge capacity vs. voltage curves of the N-u-SCC-2 electrode during the 1st, 10th, 50th, and 100th cycles. **d** Galvanostatic charge–discharge cycle of the N-u-SCC-2 electrode at a current density of 0.2 A g^−1^. **e** Cycling performance of the N-u-SCC-2 electrode at a current density of 0.5 A g^−1^

Figure 6a shows the CV curves of the N-u-SCC-2 electrode. The peak observed at ~ 0.87 V during the first cycle can be attributed to the reduction of SnO_2_ to metallic Sn or SnO and the formation of solid–electrolyte interface (SEI) layers, which accounted for the disappearing of this peak in the subsequent cycles. The relatively large area of this peak indicates that a large amount of reaction/layer was formed. The peaks observed at around 0.3 and 0.2 V in the subsequent cycles correspond to a series of Li–Sn alloying reactions. The anodic peaks at 0.50, 0.61, 0.73, and 0.78 V can be attributed to the Li_*x*_Sn → Sn dealloying reactions. This is consistent with the differential charge capacity versus voltage curves obtained in the subsequent cycles. The broad peaks at 1.25 and 2.06 V correspond to the reversible oxidation of metallic Sn to SnO and SnO_2_, respectively. The peaks remained stable and could be clearly observed during the 4th cycle, demonstrating the high reversibility of the conversion reactions. After the first cycle, the curves showed similar peak patterns, indicating that the stability of the electrode increased gradually. Figure 6b shows the first three discharge/charge curves of the N-u-SCC-2 electrode at a current density of 0.2 A g^−1^. The initial discharge capacity of the electrode was 1365.2 mAh g^−1^. Similar curves were obtained in the subsequent cycles. This attests the high ICE and excellent cycling performance of the electrode. For unveiling the reversible nature of the conversion reaction, the differential charge capacity versus voltage curves of the N-u-SCC-2 electrode at the 1st, 10th, 50th, and 100th cycles were obtained (Fig. 6c). The peaks observed over the potential range of 0.01–1.0 V correspond to the dealloying reactions (Li_*x*_Sn to Sn), while the peaks at 1.0 to − 2.4 V correspond to the conversion reactions (Sn to SnO or SnO_2_) after 100 cycles. The peaks (integral intensities and potential positions) corresponding to the conversion reactions remained the same (1st and 2nd cycles) even after the 50th cycle. These results demonstrate the high reversibility of the conversion reaction.

The N-u-SCC-2 electrode showed a reversible discharge capacity of ~ 975 mAh g^−1^ after 100 cycles (Fig. 6d) at a current density of 0.2 A g^−1^, which corresponds to a capacity retention ratio of 78.6% (when compared with the 2nd cycle). On the other hand, the N-u-SCC-1 and pure SnO_2_ electrodes exhibited much smaller reversible discharge capacities of 504 and 226.0 mAh g^−1^, respectively, after 100 cycles. The first discharge capacities of the N-u-SCC-2, N-u-SCC-1, and pure SnO_2_ electrodes were 1365.2, 990.5, and 1736.7 mAh g^−1^, respectively. This high capacity retention of the N-u-SCC-2 electrode can be attributed to its high carbon content, which provided more space for volume variations along with a larger Sn/Co contact area to inhibit the volume expansion, thus increasing the cycling life of the electrode. It should be noted that the N-u-SCC-2 electrode showed an average ICE of 82.2% because of the formation of SEI layers. This improved the reversibility of the reactions between SnO_2_ and Li. On the other hand, the N-u-SCC-1 and pure SnO_2_ electrodes showed an ICE of 68.1% and 59.7%, respectively (Fig. S6). The N-u-SCC-2 electrode exhibited smaller internal resistance than the other two electrodes, as revealed by the electrochemical impedance spectroscopy (EIS) measurements shown in Fig. S7a. The EIS measurements of the N-u-SCC-2 electrode after 100 cycles were also taken. An equivalent circuit consisting of the resistances of the electrolyte (*R*_e_), charge transfer (*R*_ct_), and constant phase elements and Warburg impedance was proposed to fit the impedance data. Low *R*_ct_ values were obtained after 100 cycles (179–32 Ω), suggesting the enhanced charge transfer kinetics (Table S4). In addition, the N-u-SCC-2 electrode showed excellent rate capability. Figure S8a shows the discharge/charge curves of the electrode at different current densities. All the curves showed similar trend with the same discharge/charge platform. Furthermore, a reversible capacity of ~ 800 mAh g^−1^ was obtained when the current density was increased to 5 A g^−1^. At the current densities of 0.2, 0.5, 1, 2, and 5 A g^−1^, discharge capacities of ~ 1500, 1240, 1090, 965, and 800 mAh g^−1^, respectively, were obtained. As the current returned to 0.2 A g^−1^, the capacity became stable, as shown in Fig. S8b. This extraordinary rate capability of N-u-SCC-2 was not due to its unique nanostructure consisting only of Co and carbon framework, but can be attributed to the oxygen vacancies, which improved the conductivity and transportation of Li^+^ [28, 31, 35–40]. The N-u-SCC-2 electrode exhibited a cycling life of up to 450 cycles and maintained a reversible capacity of ~ 760 mAh g^−1^ at the current density of 0.5 A g^−1^, as shown in Fig. 6e. After 150 cycles, the diffusion kinetics of lithium ions improved after the initial activation. The optimization of the SEI layer at the initial stage leads to capacity fading because of its breakdown and reconstruction. The cycling performance of an electrode improves after the formation of a stable SEI layer. The long-term cycling performance of the N-u-SCC-2 electrodes at relatively high current densities of 1 A g^−1^ for 200 cycles and 2 A g^−1^ for 300 cycles was also evaluated, as shown in Fig. S9. As can be observed from the figure, the electrode exhibited a desirable long-term cycling performance.

**Fig. 7:**
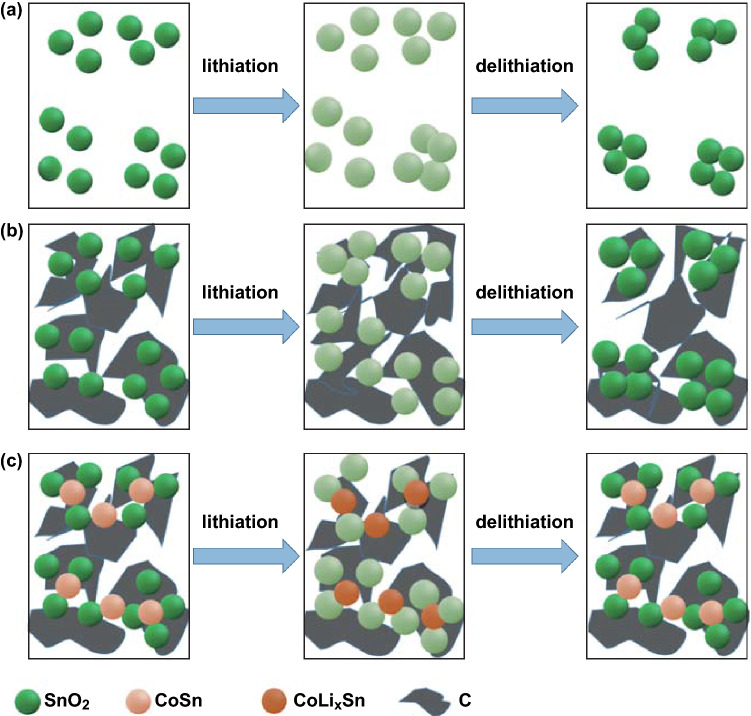
Schematic of the delithiation process of **a** pure SnO_2_, **b** SnO_2_/C, and **c** N-u-SCC

We also compared the ICE of the N-u-SCC-2 anode with that of previously reported SnO_2_/C LIB anodes. (Materials for LIBs are listed in Table S3.) The N-u-SCC-2 anode showed the highest ICE and outstanding electrochemical properties. Figure 7 shows the schematic of the delithiation process of pure SnO_2_, SnO_2_/C, and N-u-SCC electrodes. As shown in Fig. 7a, after lithiation, SnO_2_ particles became larger and the distances between them decreased. This resulted in the gradual agglomeration of SnO_2_ nanoparticles, leading to poor cycling life and low ICE of the electrode. Upon the incorporation of carbon materials like graphene and 3D frameworks (Fig. 7b), these SnO_2_ particles scattered and became more dispersive in the original state after being coated with carbon. The introduced carbon materials suppressed the volume variations of SnO_2_ particles during lithiation, provided extra space for volume variations, and served as barriers to prevent the aggregation of SnO_2_ particles so that the particles were still dispersive after delithiation. Similarly, the N-u-SCC-2 composite consisted of 3D carbon frameworks, which prevented the aggregation and volume variations of SnO_2_ particles during lithiation. However, the 3D carbon frameworks in this composite consisted of uniformly distributed Co (CoSn) particles. Co does not participate in the delithiation reactions and remains stable. This makes Co a powerful barrier to prevent the volume variations and particle agglomeration of SnO_2_. Thus, the exquisitely designed N-u-SCC-2 composite showed extraordinary electrochemical properties. Figure S10 shows the FESEM images of the ultrafine SnO_2_ particles and N-u-SCC-2 composites after 100 cycles. Pure SnO_2_ grains showed severe aggregation (Fig. S10b), while N-u-SCC-2 exhibited little aggregation and a morphology similar to that observed initially (Fig. S10d). Figure S10e, f shows the TEM images of the original N-u-SCC-2 and N-u-SCC-2 electrodes after discharging to 1.0 V. The morphology remained the same except that the latter became amorphous. These observations demonstrate the potential of the N-u-SCC-2 composite for application as an LIB anode.

## Conclusion

In summary, a novel ternary SnO_2_-Co-C nanocomposite (N-u-SCC) was successfully prepared via a simple and low-cost synthesis method. In this design, the in situ formation of Co additives from the Co-based ZIF-67 framework rendered the SnO_2_ conversion reaction highly reversible and the N-doped carbon frameworks efficiently mitigated the structural degradation of SnO_2_ while facilitating electronic transport and ionic diffusion. Accordingly, the optimized N-u-SCC electrodes exhibited excellent electrochemical performance with high ICE (average 82.2%), outstanding rate performance (800 mAh g^−1^ at 5 A g^−1^), and long-term cycling performance (~ 760 mAh g^−1^ after 400 cycles at a current density of 0.5 A g^−1^). These findings will be helpful for developing highly reversible and stable electrodes for next-generation high-performance LIBs.

## Electronic supplementary material

Below is the link to the electronic supplementary material.

Supplementary material 1 (PDF 1338 kb)
