# Supplementary material for: High Initial Reversible Capacity and Long Life of Ternary SnO2-Co-carbon Nanocomposite Anodes for Lithium-Ion Batteries
Source: Nanomicro Lett. 2019 Mar 1;11:18. doi: 10.1007/s40820-019-0246-4 (PMC7770651; doi:10.1007/s40820-019-0246-4)
Supplement: Supplementary file 1 — Supplementary material 1 (PDF 1338 kb) [file 40820_2019_246_MOESM1_ESM.pdf]

Supporting Information for

# High Initial Reversible Capacity and Long Life of Ternary $SnO_2$-Co-Carbon Nanocomposite Anodes for Lithium-Ion Batteries

Pan Deng[1], Jing Yang[1], Shengyang Li[1], Tian-E Fan[2, *], Hong-Hui Wu[3], Yun Mou[4], Hui Huang[1], Qiaobao Zhang[1, *], Dong-Liang Peng[1], Baihua Qu[1, *]

[1]Pen-Tung Sah Institute of Micro-Nano Science and Technology, and Department of Materials Science and Engineering, College of Materials, Xiamen University, Xiamen 361005, People's Republic of China

[2]College of Automation and Key Laboratory of Industrial Internet of Things and Networked Control, Ministry of Education, Chongqing University of Posts and Telecommunications, Chongqing 400065, People's Republic of China

[3]Department of Chemistry, University of Nebraska-Lincoln, NE 68588 Lincoln, United States

[4]School of Mechanical Science and Engineering, Huazhong University of Science and Technology, Wuhan 430074, People's Republic of China

*Corresponding authors. E-mail: bhqu@xmu.edu.cn (Baihua Qu); fante@cqupt.edu.cn (Tian-E Fan); zhangqiaobao@xmu.edu.cn (Qiaobao Zhang)

## Supplementary Figures and Tables

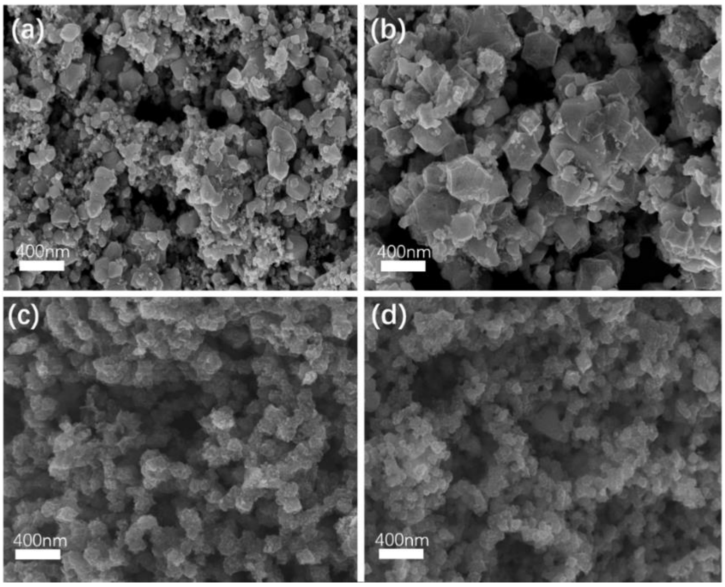


**Fig. S1** Morphology of **a** pure $SnO_2$; **b** N-c-SSC-1; **c** N-c-SSC-2; **d** N-c-SSC-3

The morphology of the commercial $SnO_2$, N-c-SSC at the ratio of 1.25 mmol-$Co(NO_3)_2{\cdot}6H_2O$ and 5mmol-2-Mi (N-c-SSC-1), N-c-SSC at the ratio of 1.25 mmol-$Co(NO_3)_2{\cdot}6H_2O$ and 10mmol-2-Mi (N-c-SSC-2), N-c-SSC at the ratio of 2 mmol-$Co(NO_3)_2{\cdot}6H_2O$ and 20mmol-2-Mi (N-c-SSC-3) were observed by scanning electron microscope (FESEM) as shown in Fig. S1. The pure $SnO_2$ particles shows irregular shape with different morphologies in Fig. S1a. After adding ZIF-67, the frameworks of ZIF-67 can be observed in Fig. S1b-d. When the ratio of $Co(NO_3)_2{\cdot}6H_2O$ and 2-Mi is 1:4, the frameworks exhibit bigger (~600 nm) compared to that (~250 nm) in Fig. S1c, d. in which the morphology is a melange of regular frameworks and irregular commercial $SnO_2$ particles, and the commercial $SnO_2$ are attached to the frameworks. It comes out quite different that much more uniform particles are obtained when the ratio is 1:8, both of Fig. S1c and S1d exhibit the same polyhedral structure, the irregular $SnO_2$ particles disappear due to the shield of the small frameworks. Also, smaller nanoparticles can be noticed on the frameworks in Fig. S1c, which doesn’t seem so obvious in Fig. S1d. This may be related to the higher carbon content.

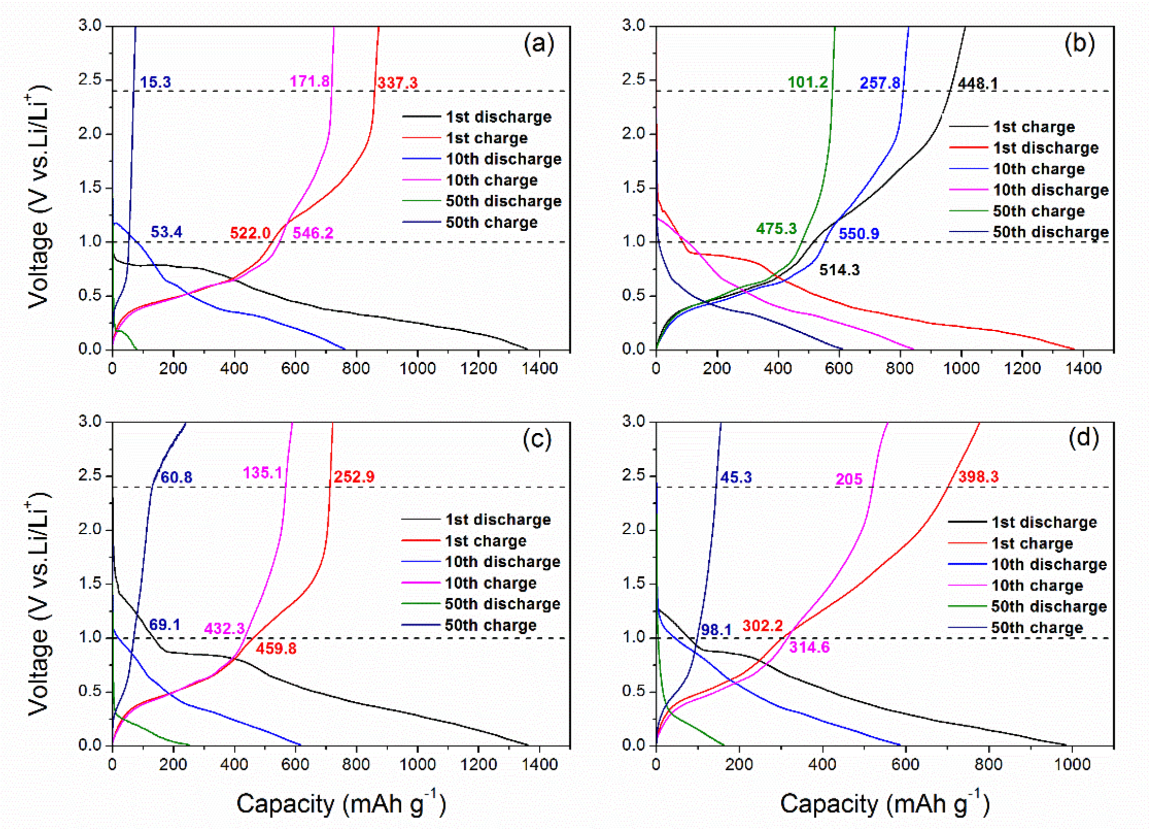


**Fig. S2** Charge and discharge curves of **a** commercial $SnO_2$, **b** N-c-SCC-1, **c** N-c-SCC-2 and **d** N-c-SCC 3 electrodes cycled between 0.01 and 3 V at 0.2 A $g^{-1}$

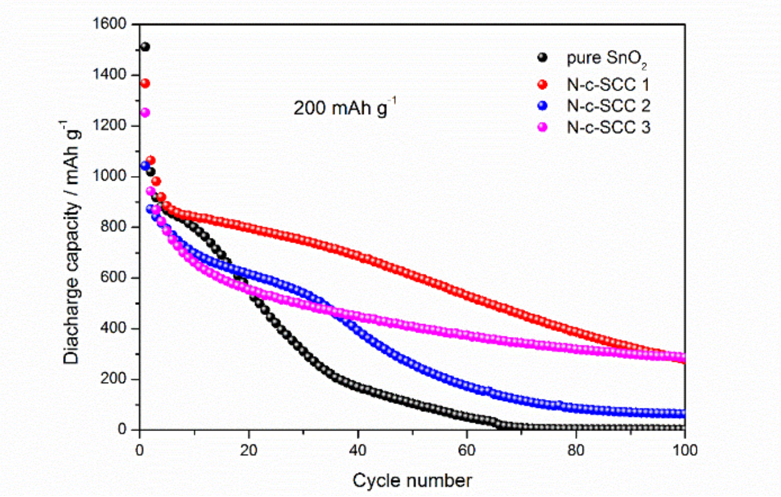


**Fig. S3** Cycling performance of the commercial $SnO_2$ and N-c-SCC electrodes at a current density of 0.2 A $g^{-1}$

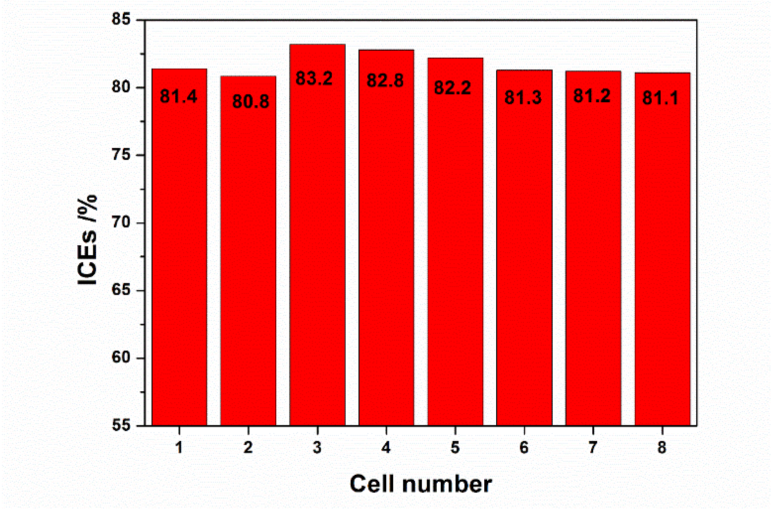


**Fig. S4** Statistical ICE of some typical cells

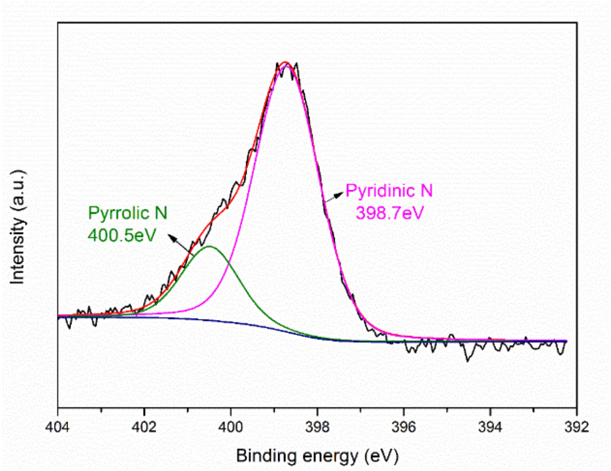

**Fig. S5** High-resolution XPS spectra of N1s in N-u-SCC-2 composite

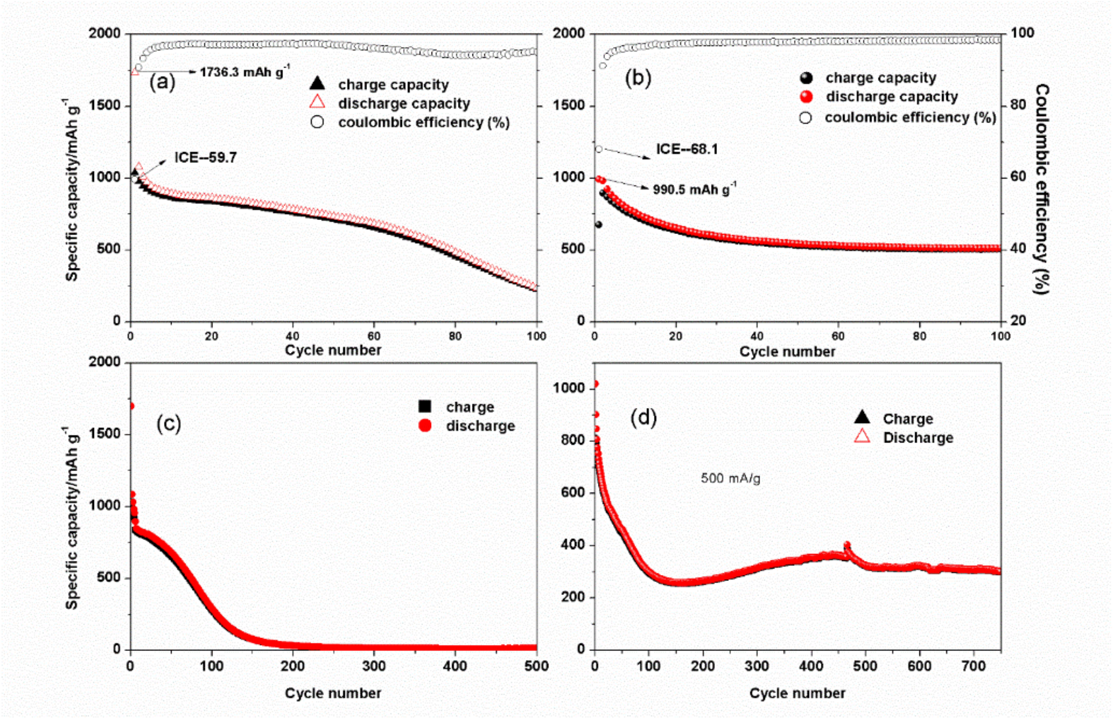


**Fig. S6** Cycling performance of **a** ultrafine $SnO_2$ electrode at a current density of 0.2 A $g^{-1}$, **b** N-u-SCC-1 electrodes at a current density of 0.2 A $g^{-1}$, **c** ultrafine $SnO_2$ electrode at a current density of 0.5 A $g^{-1}$, **d** N-u-SCC-1 electrodes at a current density of 0.5 A $g^{-1}$

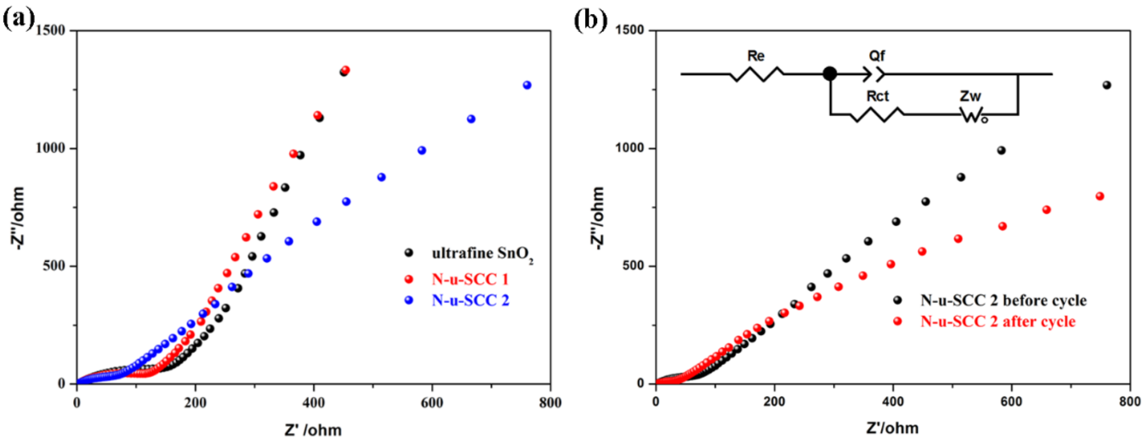


**Fig. S7 a** Nyquist plots of the three ultrafine $SnO_2$, N-u-SCC-1, N-u-SCC-2 electrodes. **B** Nyquist plots of N-u-SCC-2 electrodes before and after 100 cycles and the proposed equivalent circuit to fit the impedance data

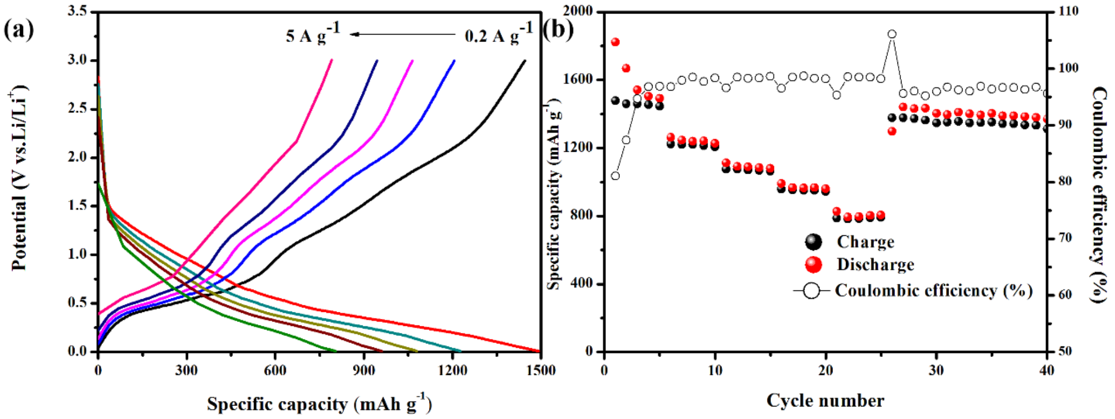


**Fig. S8 a** Discharge/charge curves at different current densities of the N-u-SCC-2 electrode. **b** Rate performance of the N-u-SCC-2 electrode

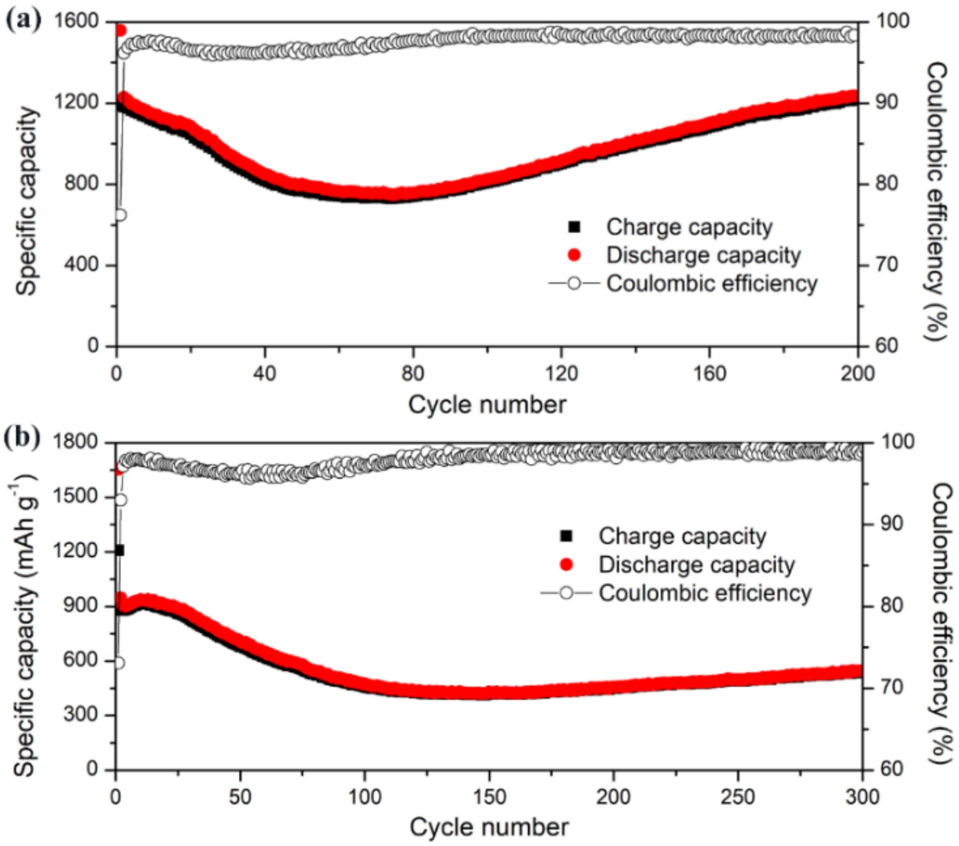


**Fig. S9** Cycling performance of the N-u-scc-2 electrode at a current density of **a** 1 A $g^{-1}$ and **b** 2 A $g^{-1}$

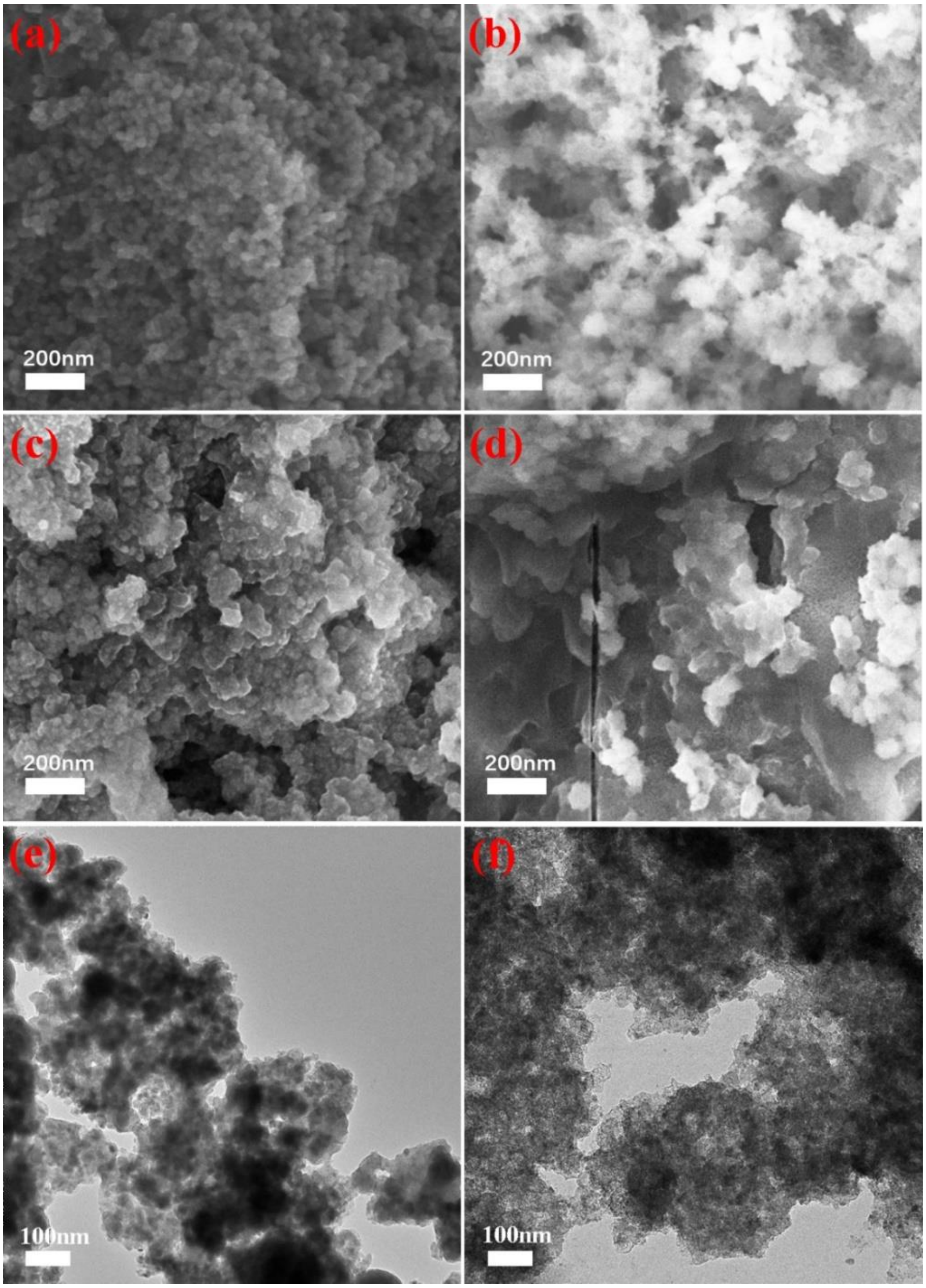


**Fig. S10** FESEM image of pure ultrafine $SnO_2$ **a** before and **b** after 100 cycled, of N-u-SCC 2 **c** before and **d** after 100 cycled. TEM images of N-u-SCC 2 **e** before and **f** after discharging to 1.0 V

**Table S1** Part of basic facts of the N-c-SCC composites

| Defined name | Mole dosage of $Co(NO_3)_2 \cdot 6H_2O$ (mmol) | Mole dosage of 2-Melm (mmol) | ICE (%) |
|---|---|---|---|
| N-c-SCC-1 | 1.25 | 5 | |
| N-c-SCC-2 | 1.25 | 10 | 72.5 |
| N-c-SCC-3 | 2.5 | 20 | |

Table 1 lists synthetic formula and ICE about the three N-c-SCC composites.

**Table S2** EDS results of the two N-u-SCC composites

| N-u-SCC-1 | | | N-u-SCC-2 | | |
|---|---|---|---|---|---|
| element | Atomic fraction (%) | Mass fraction (%) | element | Atomic fraction (%) | Mass fraction (%) |
| C | 17.21 | 5.43 | C | 23.97 | 9.68 |
| N | 19.24 | 6.93 | N | 21.23 | 9.80 |
| O | 25.73 | 11.00 | O | 33.70 | 18.46 |
| Co | 19.02 | 25.12 | Co | 6.63 | 11.23 |
| Sn | 18.80 | 51.53 | Sn | 14.48 | 50.83 |

**Table S3** Comparison of the ICEs and electrochemical properties of N-u-SCC-2 with some reported $SnO_2$/C anode materials for LIBs

| Type of composite | ICE (%) | Current density (A $g^{-1}$) | Capacity (mAh $g^{-1}$) | cycles | Potential window | Year | Refs. |
|---|---|---|---|---|---|---|---|
| $SnO_2$NC@N-RGO | 61.3 | 0.5 | 1346 | 500 | 0.005-3 V | 2013 | [1] |
| Bowl-like $SnO_2$@C | 68.4 | 0.4 | 963 | 100 | 0.005-3.0 V | 2014 | [2] |
| Core-shell $SnO_2$/C | 69.3 | 0.1 | 750 | 100 | 0.01-3 V | 2015 | [3] |
| $SnO_2$@N-CNF | 69.2 | 0.1 | 754@1 A $g^{-1}$ | 300 | 0.01-3 V | 2016 | [4] |
| $SnO_2$/NC | 51 | 0.5 | 491 | 100 | 0.01-2 V | 2016 | [5] |
| $SnO_2$/Co@C | 66 | 0.2 | 800 | 100 | 0.01-2.5 V | 2017 | [6] |
| PDA-coated $SnO_2$ | 61.3 | 0.16 | ~1200 | 300 | 0.01-3 V | 2017 | [7] |
| $SnO_2$-Mn-G | 76.2 | 0.2 | 850 | 400 | 0.01-3 V | 2017 | [8] |
| Porous $SnO_{2-б}$/C | 74.3 | 0.1 | 543@1 A $g^{-1}$ | 1000 | 0.01-3 V | 2018 | [9] |
| NuSCC-2 | 82.2 | 0.2 | 975@0.2 A $g^{-1}$<br>760@0.5 A $g^{-1}$ | 100<br>400 | 0.01-3 V | Our work | |

**Table S4** EIS fitting results of N-u-SCC-2 electrode before and after cycling

| Element | Value 1 (before cycling) | Value 2 (after 100 cycling) |
|---|---|---|
| *R*e | 0.703 | 2.785 |
| *R*ct | 179 | 32.1 |
| *Z*w-R | 1.745 | 3079 |
| *Z*w-T | 40.23 | 5.412 |
| *Z*w-P | 0.867 | 0.640 |
| *Q*ct-T | 0.0003 | 9.11E-05 |
| *Q*ct-P | 0.526 | 0.646 |

## Supplementary References


[1] X. Zhou, L. J. Wan, Y.G. Guo, Binding $SnO_2$ nanocrystals in nitrogen-doped graphene sheets as anode materials for lithium-ion batteries. Adv. Mater. **25**(15), 2152-2157 (2013). https://doi.org/10.1002/adma.201300071

[2] J. Liang, X.Y. Yu, H. Zhou, H.B. Wu, S. Ding, X.W. Lou, Bowl-like $SnO_2$ @carbon hollow particles as an advanced anode material for lithium-ion batteries. Angew. Chem. Int. Ed. **53**(47), 12803-12807 (2014). https://doi.org/10.1002/anie.201407917

[3] D. Zhou, W.L. Song, L.Z. Fan, Hollow Core-Shell $SnO_2$/C Fibers as Highly Stable Anodes for Lithium-Ion Batteries. ACS Appl. Mater. Interfaces **7**(38), 21472-21478 (2015). https://doi.org/10.1021/acsami.5b06512

[4] L. Xia, S. Wang, G. Liu, L. Ding, D. Li, H. Wang, S. Qiao, Flexible $SnO_2$/N-Doped Carbon Nanofiber Films as Integrated Electrodes for Lithium-Ion Batteries with Superior Rate Capacity and Long Cycle Life. Small **12**(7), 853-859 (2016). https://doi.org/10.1002/smll.201503315

[5] X. Zhou, L. Yu, X.W. Lou, Formation of Uniform N-doped Carbon-Coated $SnO_2$ Submicroboxes with Enhanced Lithium Storage Properties. Adv. Energy Mater. **6**(14), 1600451 (2016). https://doi.org/10.1002/aenm.201600451

[6] Q. He, J. Liu, Z. Li, Q. Li, L. Xu, B. Zhang, J. Meng, Y. Wu, L. Mai, Solvent-Free Synthesis of Uniform MOF Shell-Derived Carbon Confined $SnO_2$/Co Nanocubes for Highly Reversible Lithium Storage. Small **13**(37), 1701504 (2017). https://doi.org/10.1002/smll.201701504

[7] B. Jiang, Y. He, B. Li, S. Zhao, S. Wang, Y.B. He, Z. Lin, Polymer-Templated Formation of Polydopamine-Coated $SnO_2$ Nanocrystals: Anodes for Cyclable Lithium-Ion Batteries. Angew. Chem. Int. Ed. **56**(7), 1869-1872 (2017). https://doi.org/10.1002/anie.201611160

[8] R. Hu, Y. Ouyang, T. Liang, X. Tang, B. Yuan, J. Liu, L. Zhang, L. Yang, M. Zhu, Inhibiting grain coarsening and inducing oxygen vacancies: the roles of Mn in achieving a highly reversible conversion reaction and a long life $SnO_2$–Mn–graphite ternary anode. Energy Environ. Sci. **10**(9), 2017-2029 (2017). https://doi.org/10.1039/C7EE01635B

[9] R. Jia, J. Yue, Q. Xia, J. Xu, X. Zhu, S. Sun, T. Zhai, H. Xia, Carbon shelled porous $SnO_{2-\delta}$ nanosheet arrays as advanced anodes for lithium-ion batteries. Energy Storage Mater. **13**, 303-311 (2018). https://doi.org/10.1016/j.ensm.2018.02.009
